# Image-based dissolution analysis for tracking the surface stability of amorphous powders

**DOI:** 10.5599/admet.839

**Published:** 2020-07-13

**Authors:** Jernej Štukelj, Mikael Agopov, Jouko Yliruusi, Clare J. Strachan, Sami Svanbäck

**Affiliations:** 1 Division of Pharmaceutical Chemistry and Technology, University of Helsinki, Viikinkaari 5E, 00790 Helsinki, Finland; 2 The Solubility Company Oy, Viikinkaari 4, 00790 Helsinki, Finland

**Keywords:** Solubility, Stability, Crystallization, Amorphous, Single-Particle Analysis, SPA

## Abstract

Poor solubility of crystalline drugs can be overcome by amorphization – the production of high-energy disordered solid with improved solubility. However, the improved solubility comes at a cost of reduced stability; amorphous drugs are prone to recrystallization. Because of recrystallization, the initial solubility enhancement is eventually lost. Therefore, it is important to understand the recrystallization process during storage of amorphous materials and its impact on dissolution/solubility. Here, we demonstrate the use of image-based single-particle analysis (SPA) to consistently monitor the solubility of an amorphous indomethacin sample over time. The results are compared to the XRPD signal of the same sample. For the sample stored at 22 °C/23% relative humidity (RH), full crystallinity as indicated by XRPD was reached around day 40, whereas a solubility corresponding to that of the γ crystalline form was measured with SPA at day 25. For the sample stored at 22 °C/75% RH, the XRPD signal indicated a rapid initial phase of crystallization. However, the sample failed to fully crystallize in 80 days. With SPA, solubility slightly above that of the crystalline γ form was measured already on the second day. To conclude, the solubility measured with SPA directly reflects the solid-state changes occurring on the particle surface. Therefore, it can provide vital information – in a straightforward manner while requiring only minuscule sample amounts – for understanding the effect of storage conditions on the dissolution/solubility of amorphous materials, especially important in pharmaceutical science.

## Introduction

In pharmaceutical industry, solid-state form selection of an active pharmaceutical ingredient (API) is an important step in the development of a solid dosage form. The solid-state form governs the processability, stability and bioavailability of the final product. Thus, poorly soluble crystalline APIs can be replaced by their respective, more soluble, amorphous forms [1-3]. However, an increase in apparent solubility, and consequently bioavailability, comes at a price of instability. Therefore, monitoring and understanding of amorphous form instability – recrystallization – is crucial for optimal product design.

The non-steroidal-anti-inflammatory drug indomethacin has been widely used as model drug to study recrystallization behaviour with the first studies dating all the way back to the 1970s [4, 5]. Stability studies looking at amorphous indomethacin can be divided in two groups: amorphous stability during storage and amorphous stability during dissolution testing.

The behaviour of amorphous indomethacin during storage is governed by temperature and humidity [6–8]. The two parameters determine the direction of crystallization of amorphous indomethacin prepared by cooling the melt; at 30 °C, the metastable α form predominantly crystallizes at high humidity (>56% relative humidity (RH)) and the thermodynamically stable γ form predominantly crystallizes at low humidity (<43% RH) [6]. Water acts as a plasticizer and thus, with an increase in humidity, the glass transition (*T*_g_) of amorphous indomethacin decreases. Temperatures below the *T*_g_ favour formation of the γ form and temperatures above *T*_g_ favour formation of the α form. Furthermore, crystallization may also be considered from a surface versus bulk perspective: for quench-cooled amorphous films, V. Andronis *et al*. reported 30 °C/11% RH as a limit below which crystallization was surface-initiated and 30 °C/21% RH as a limit above which crystallization was bulk-initiated [6]. In addition, Wu et al. reported surface crystallization being two orders of magnitude higher compared to bulk crystallization for amorphous films stored at 40 °C [8].

When exposed to dissolution medium, the amorphous solid undergoes solid-state transformation via two pathways: a direct recrystallization of the solid and/or a solution mediated crystallization from the supersaturated solution [9]. D. Alonzo et al. reported that crystallization from supersaturated solution was the main mechanism reducing the solubility of amorphous indomethacin. However, the adsorption of water onto the exposed amorphous surface increases molecular mobility and thus potentially promotes the surface crystallization rate [6, 10]. The dissolution studies in flow-through chambers coupled with Raman probes enabled the monitoring of dissolution rate and crystallization, revealing the dependency of dissolution rate on processing history of the amorphous solid [11, 12].

Priemel et al. studied amorphous tablets stored at 30 °C/23% RH with differential scanning calorimetry (DSC), X-ray powder diffraction (XRPD) and Fourier-transform infrared (FTIR) spectroscopy and correlated the results of the analytical techniques to the dissolution performance of tablets after five days of storage [13]. The dissolution is a purely surface phenomenon, and thus correlating it with the results provided by the bulk analytic techniques can be challenging. Novakovic *et al*. employed nonlinear optics to study surface crystallization and dissolution of indomethacin tablets (300 mg) stored at 30 °C/23% RH and 30 °C/75% RH for 1, 2, 7 and 22 days [14]. A trend towards decreased dissolution rate with increased storage time was observed. Furthermore, Novakovic et al. reported a delay in crystallization onset during storage and multiple factors affecting dissolution of amorphous indomethacin tablets (500 mg) as a result of the polymer coating [15].

By now, one should note the amount of research conducted with amorphous indomethacin is quite considerable. However, the studies that have investigated the dissolution/solubility enhancement of amorphous indomethacin – or any other amorphous drug – throughout the storage time are scarce. In our opinion, this is due to the following factors: substantial amount of drug compound needed and challenges for analytical methods to detect the changes in dissolution/solubility of the originally amorphous sample [16]. Nevertheless, these studies are highly important as surfaces dictate the dissolution behaviour of pharmaceutical compounds and by understanding the processes occurring on the surfaces we can better predict their *in vivo* performance.

**Figure 1.**
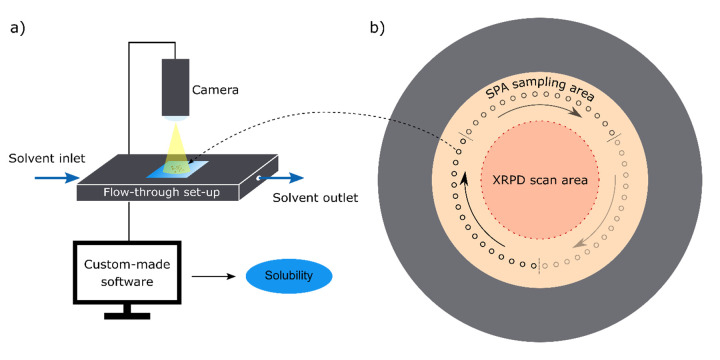
**(a)** Schematic of the single-particle analysis (SPA) setup. **(b)** Schematic of the low-scatter-background holder and sampling protocol used in the study. The middle area (red) was scanned, on the measuring days, with the XRPD and was not altered throughout the course of the study. The SPA sampling area was between the XRPD scan area and the flush aluminium surface of the holder (grey). On the measuring day, three samples from this area were taken with a spatula from opposite sides of the holder starting with day zero at the three dashed lines and then moving clockwise with each consecutive measuring day. In that way, the sampling area distribution was maximized and no spot was sampled more than once.

With the emergence of the image-based single-particle analysis (SPA) for determining the solubility (Figure 1a), these two limiting factors above can be easily tackled [17]. The SPA methodology requires minuscule sample amounts (<100 μg), short equilibration time and it has already proven successful in directly measuring the amorphous solubility, also referred to as apparent solubility, of several compounds [18]. In this study, we continuously monitor the XRPD signal and the solubility, employing SPA, of amorphous indomethacin stored at two different ambient conditions for 80 days.

## Experimental

### Materials

Indomethacin (γ form) was received from Orion Pharma, Espoo, Finland. The α form was prepared by dissolving 3 g of indomethacin in 20 mL of EtOH and heated up to 70 °C. Upon complete dissolution, the system was kept at the set temperature for an additional 5 min and then rapidly cooled down in an ice bath. The formed slurry was filtered and dried in a vacuum oven at 70 °C for 1 h. Amorphous indomethacin was prepared by melting the γ form on a hot plate at 165 °C. After visual confirmation of complete melting, the melt was kept on a hot plate for additional 30 seconds. After 30 seconds, the melt was immediately cooled down on an aluminium heat sink. It has been shown previously that this preparative technique does not cause significant sample degradation [19–21]. The quench cooled melt was then stored over phosphorus pentoxide at room temperature for one hour. After one hour, the amorphous indomethacin was gently ground with a mortar and pestle. A small amount of the sample was taken for the DSC analysis and the remaining amount was halved and placed into two XRPD holders with a low-scatter background (Figure 1b).

### Storage conditions

One of the two sample holders with the amorphous indomethacin was stored at 23% RH and the other at 75% RH. Both samples were kept at 22±0.5 °C.

### Differential Scanning Calorimetry (DSC)

DSC measurements were conducted to characterize the solid-state of crystalline and amorphous samples. Samples (2 to 5 mg) were packed into standard aluminium crucibles (40 μL) with pierced lids. The samples were analysed with a DSC823e instrument (Mettler-Toledo, Greifensee, Switzerland) equipped with a cooling system (Julabo FT 900, Seelbach, Germany). Nitrogen (50 mL/min) was used as a purge gas. Equilibration at 25 °C for 3 minutes was followed by linear heating with a heating rate of 10 °C/min. Measurements were made in triplicate. Thermal events were analysed using the STARe software (Mettler-Toledo, Greifensee, Switzerland).

### X-ray powder diffraction (XRPD)

XRPD diffractograms were recorded to characterize the solid-state of the crystalline and amorphous samples. Moreover, XRPD was used to follow the solid-state changes of the two amorphous samples over time (Figure 1b). An Aeris diffractometer (Malvern Panalytical B.V., Almelo, Netherlands) using Cu Kα radiation (*λ* = 1. 1.540598 Å) and a divergence slit of 0.76 mm was used. Samples were placed on a low-scatter-background holder and measured with a step size of 0.0108664° at 40 kV and 8.0 mA from 5° to 35° (2θ). The XRPD measurements of the two amorphous samples were carried out on the following days: 0, 2, 4, 7, 10, 14, 17, 21, 25, 29, 32, 38, 44, 53, 60 and 80. The height of the peaks at 11.6°, 16.7°, 21.8° and 26.6° of the 2θ angle was used to quantify the solid-state change that occurred. The height of each peak was divided by the height of the silicon peak (28.4°) measured on the same day to correct the response for the fluctuation of the X-ray beam. The silicon peak was measured from the silicon plate provided by Malvern Panalytical. The four corrected peak height values were then averaged in order to obtain a single quantifying value.

### Single-particle analysis (SPA)

Sampling of the two amorphous samples stored at 23% and 75% RH was carried out as presented in Figure 1. On each of the measuring days (0, 2, 4, 7, 10, 14, 17, 21, 25, 29, 32, 38, 44, 53, 60 and 80) three samples were extracted from opposite sides of the XRPD sample holder and never from the same location twice. The XRPD scan area was left intact in order for sampling not to affect the XRPD signal. An aqueous solution of HCl pH 2.0 was used as a solvent. Separately, crystalline samples of α and γ indomethacin were also measured with the SPA method.

The SPA methodology has already been previously described by Svanbäck et al. and Štukelj *et al*. [17, 18, 22]. Shortly, the SPA is comprised of two prime components – the flow-through setup and the custom-made analysis software (Figure 1a). The flow-through setup is capable of immobilizing the drug particles under steady flow. Immobilized particles are imaged as they dissolve – reduce in size. In this study, each measurement took roughly 5 minutes. Captured images of the particles are analysed using the custom-made software, which tracks the reduction in particle morphology throughout the course of a measurement. The decrease in mass over time or dissolution rate (d*m*/d*t*) under sink conditions (when the bulk concentration (*C*_b_) can be assumed to be zero) is, according to Noyes and Whitney (eq. 1), directly proportional to the equilibrium solubility (*C*_s_) of a compound. The setup-dependent-transport-rate constant (*k*) is determined as described by Svanbäck *et al*. [23]. Furthermore, if the compound is in its most stable crystalline form, the determined equilibrium solubility corresponds to the thermodynamic equilibrium solubility of a compound. On the other hand, for a purely amorphous sample, in the absence of crystallisation during the SPA measurement, the measured solubility is actually the amorphous solubility – the maximum drug concentration in solution upon dissolution of an amorphous solid. The approach of measuring amorphous solubility using the SPA technology was recently validated by Štukelj *et al*. (2019), with the amorphous solubility of five diverse compounds being assessed by SPA and two orthogonal methods: standardized supersaturation and precipitation, and theoretical estimation based on thermal analysis [18].


(1)

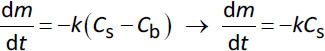



## Results and Discussion

### Characterization of the initial solid-state form

**Figure 2.**
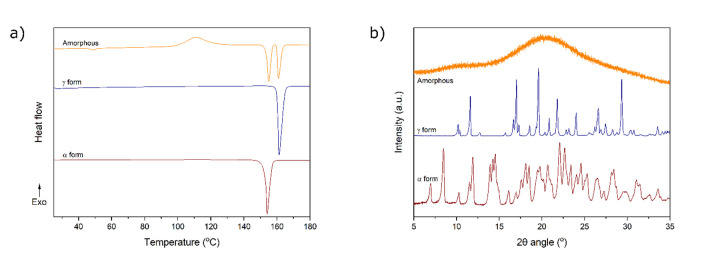
**(a)** Thermograms of α, γ and amorphous indomethacin samples. **(b)** Diffractograms of α, γ and amorphous indomethacin samples.

The DSC thermograms of the amorphous and two crystalline indomethacin forms are presented in Figure 2a. The amorphous thermogram exhibits a glass transition mid-point at 44.2 ± 1.0 °C, a crystallization onset at 99.5 ± 0.2 °C, a melting peak onset corresponding to the α form at 153.2 ± 0.1 °C and a melting peak onset corresponding to the γ form at 159.8 ± 0.1 °C. Crystallization of freshly-prepared melted and cooled amorphous indomethacin during heating to both these forms has been observed earlier [13, 24]. The thermogram of the γ form exhibits a melting peak at 159.8 ± 0.2 °C and the thermogram of the α form exhibits a melting peak at 152.7 ± 0.2 °C. The XRPD diffractogram of the amorphous sample exhibits a distinctive amorphous halo and is devoid of any peaks that would indicate crystallinity (Figure 2b) [13]. The diffractograms of the α and γ crystalline samples match the predicted diffractograms of the respective forms (INDMET02 and INDMET01) from the Cambridge Structural Database.

### Solubility of α and γ indomethacin

The intrinsic solubility of α and γ indomethacin measured using the SPA method is compared to the intrinsic solubility of these two forms as assessed using the orthogonal methods in Table 1. The intrinsic solubility of the α form was measured previously by Surwase *et al*. using a shake-flask method [20]. The intrinsic solubility of the γ form was measured by Štukelj et al. using a μDISS profiler™ [18]. The obtained values are in good agreement and within the generally accepted average uncertainty in experimental solubility measurements of 0.6 log units [25].

### Tracking the solid-state changes and amorphous solubility over time

**Figure 3.**
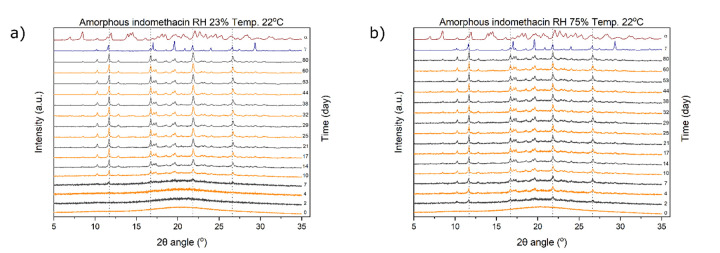
XRPD diffractograms of the amorphous samples stored at (a) 22 °C/23% RH and (b) 22 °C/75% RH. Dashed lines indicate the γ form XRPD peaks used for quantification: 11.6°, 16.7°, 21.8° and 26.6°. Experimentally recorded diffractograms of α and γ indomethacin are plotted for comparison.

The XRPD diffractograms recorded over time of the two amorphous samples stored at 22 °C/23% RH and 22 °C/75% RH are presented in Figure 3. Crystallization started sooner at 75% RH than at 23% RH, as peaks of crystallinity can already be seen on the second day (Figure 3b). In contrast, at 23% RH the first peaks of crystallinity can be only observed at day 7 (Figure 3a). Both samples predominantly crystallized into the γ form; the differences in relative peak intensities for the stored samples versus the reference γ form diffractogram most likely arise due to crystal habit (or possibly preferred crystal orientation) differences associated with the different crystal growth conditions. The presence of a minor amount of the α form is indicated by a small peak appearing at 8.5°. A study with amorphous indomethacin tablets prepared via the melt and stored at 30 °C/23% RH and 30 °C/75% RH was performed by Novakovic *et al*. [26]. The researchers stored tablets for 22 days and observed transformation into the γ form at 23% RH and the α form at 75% RH. The transformation, however, was not exclusive, as regions that could be associated with the α form were also detected at 23% RH. In contrast, Patterson *et al*. obtained a predominantly γ sample with some α form present when they stored quench cooled amorphous indomethacin at 30 °C/75% RH [21]. This ostensibly stochastic event could have been the result of other factors. Besides temperature and humidity, preparation method and parameters can also influence the resulting polymorphic form [24].

**Figure 4.**
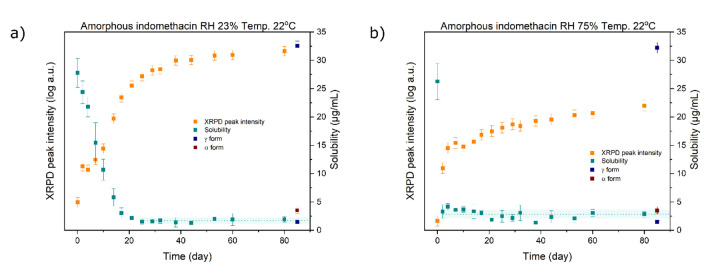
**(a)** Plot of the XRPD quantified signal and amorphous solubility of the sample stored at 22 °C/23% RH. Dotted line represents the average plateau solubility (1.69 ± 0.58 μg/mL). **(b)** Plot of the XRPD quantified signal and amorphous solubility of the sample stored at 22 °C/75% RH. Dotted line represents the average plateau solubility (2.04 ± 0.79 μg/mL).

To quantify the solid-state changes of the amorphous samples, four distinctive peaks characteristic of the γ form at 11.6°, 16.7°, 21.8° and 26.6° 2θ were selected and are indicated by the dashed lines in Figure 3. The resulting values were averaged and the log value of the average plotted against time (Figure 4). In 80 days, the amorphous sample stored at 23% RH almost fully converted into the γ form (Figure 4a). In contrast, full conversion of the sample stored at 75% RH, as measured by the averaged peak intensity, did not occur despite the crystallization starting sooner than for the sample stored at 23% RH. The presence of the elevated XRPD baseline, indicating amorphousness, at the end of the study (Figure 3b) could be explained by one, or a combination, of two possibilities: i) rapid nucleation at higher humidity resulted in the formation of nanocrystals, which due to their small size and many defects still appeared partially amorphous, and ii) the presence and morphology of crystals at the particle surface may have acted as a mechanical barrier for further crystal growth in the particle core. When interpreting the XRPD results, one must also keep in mind that the penetration depth of X-rays is in the order of several hundred μm and that results may significantly vary if analysed with a different technique [13].

Sampling for the SPA measurements was conducted as depicted in Figure 1b. Three measurements of the amorphous solubility of each sample conducted on the same day were averaged and plotted against time (Figure 4). For the sample stored at 23% RH, the amorphous solubility gradually decreased and reached that of the γ crystalline form (1.69 ± 0.58 μg/mL) at day 25. In contrast, the XRPD signal reached a plateau only around day 40 (Figure 4a). Thus, even prior to the sample being fully crystalline based on the XRPD measurements, its dissolution rate and solubility were already fully governed by the crystalline form. This can be explained by the surface crystallizing faster than the bulk [8]. Priemel *et al*. have observed that only the surface of freshly prepared amorphous tablets crystallized during dissolution testing [13]. In contrast, the whole tablet crystallized during dissolution when it was stored for 5 days at 30 °C/23% RH. The researchers explained the observation by a denser crystalline layer forming during dissolution, which acted as a mechanical barrier for further solution-mediated crystallization, versus a less dense crystalline layer forming during storage, which eventually promoted the migration of crystallization further into the tablet core. Consequently, the dissolution rate of amorphous indomethacin tablets stored for five days matched the dissolution rate of the γ form.

For the sample stored at 75% RH, the solubility sharply drops already on the second day and then fluctuates around 2.04 μg/mL – slightly above the solubility of the reference γ form, but below the solubility of the α form measured in this study (3.5 ± 0.6 μg/ml). We have earlier reported the solubility of the α indomethacin form being roughly 3 times higher compared to the solubility of the γ form [22]. However, the higher solubility of the amorphous sample stored at 75% RH measured over time in this study is most likely the result of one or both of the following phenomena: i) the presence of minor amounts of the α form in the sample, and ii) differences in surface morphology, including crystal habit, size and crystal defects, due to nanocrystals forming during storage, as observed by Priemel *et al*. [13], versus single micro-scale crystals of the reference γ form.

The sharp drop in solubility on the second day, on the other hand, may be the result of two coinciding factors: i) grinding resulted in the formation of crystallization nuclei on the surface of the particles, which were undetectable with the XRPD [21], and ii) during storage, adsorbed water on the surface increased the molecular mobility and decreased the *T*_g_ of the amorphous indomethacin thus further facilitating the surface crystallization [6, 10]. Once exposed to the dissolution medium, even if the surface was not yet fully crystalline, it was certainly very prone to rapid crystallization.

## Conclusions

In this study, we have demonstrated the suitability of the SPA technology for tracking the stability/solubility of an initially amorphous sample during storage. The short equilibration time and low sample consumption of the method enabled – for the first time – continuous observation of solubility of a particulate amorphous sample as opposed to tablets which have conventionally been required.

The results for the amorphous indomethacin stored at 22 °C/23% RH indicate a gradual decrease in solubility towards the intrinsic solubility of the γ form. The y form solubility was reached at day 25, which was roughly 15 days prior to XRPD detecting a fully crystalline sample.

For the sample stored at 22 °C/75% RH, the XRPD signal initially indicated fast crystallization. However, the sample did not fully crystallize in 80 days. In contrast, on the second day, SPA detected a rapid decrease in solubility towards a value slightly above the solubility of the γ form. The slightly higher solubility was most likely measured due to the presence of crystals with different morphology, nanocrystals, and some associated residual disorder on the surface compared to single crystals of the reference γ form.

The stability of an amorphous material approached from the solubility perspective, as enabled with SPA, directly reflects the changes occurring on particle surfaces that dictate the dissolution behaviour. This straight-forward and sensitive approach could provide crucial information for better understanding of the link between stability of amorphous, and also other, materials, their solubility and *in vivo* performance, which is especially important in pharmaceutical science.

## Figures and Tables

**Table 1.** Intrinsic solubilities of α and γ indomethacin measured with the SPA method compared to previously determined intrinsic solubility values using the shake-flask (α form) and μDISS Profiler™ (γ form) methods.

	Intrinsic solubility (μg/mL)	
	Shake-flask/μDISS profiler™	SPA
α form	2.4±0.2^a^	3.5±0.6
γ form	1.3±0.3^b^	1.5±0.2

^a^Reference [20].

^b^Reference [18]
